# Animal Language

**Published:** 1885-08

**Authors:** 


### Animal Language.

This subject of animal language interests me more and more, for I find it is not merely a power they have, but a very cultivable power. If you will talk with the creatures in your back yard, they soon get not only to understand your general meaning, but to reply with modifications of sound that are meant for you in particular.

One or two hens in a flock will show particular aptitude in telling you what they think. Some horses have, or can have, an octave of sounds, and express very nice gradations of dislike and pleasure, even up to defined laugh. Other animals laugh, notably birds, with articulate sounds.

Some years since, I owned a horse, with which I undertook to drive to a neighboring town over the hills in winter. A spot of hidden ice suddenly tripped her, and for a time it was impossible for her to get up. But by efforts that entirely exhausted me, I finally got her on foot again. She never forgot it. But my approach to the stable was invariably welcomed by cordial neighs, and that not sufficing, she would put her head affectionately on my shoulder or under my arm.

On another occasion a pet Morgan called me, while I was engaged fifty rods from the barn, with loud and persistent calls that I instantly understood meant trouble. Going hastily to the stables, I found the cows had broken down a door, and were capable of doing mischief. As soon as I approached, the horse gave a satisfied whinny, followed by a long sigh of relief, and went to eating very quietly.

But for real vocalization, the fowls surpass both animals and birds. I have had one rooster that had a remarkable power of laughing. No one could possibly hear him, without doing as he would when Gough laughs—laugh with him.

Now a dog has a diffused laugh all over, but especially concentrates a chuckle in his tail. The dog's tail is the most cultured tail among animals. It not only laughs, but it talks.

The tail of a cow has no more sense than an ox-gad, and a horse's tail is not much wiser. They even lash flies in a loose way that shows they don't know, except in a general way, where the fly is. It was necessary that men should lose such a brainless appendage. As the brain increases, the tail decreases, and laughing and talking go over to the brain.

In studying animal language, we must study, not only their power of adaptibility to us, but our power of adaptation to them. That is, having ourselves evolved our language from the sign and emotion stage, there is still in us a capacity for going back to those creatures that have not yet come up to an intelligent language ; we can put forth an effort to meet them half way.

Prof. Bartholomew, with his trained horses, talks with them in equine language. It needs to drop words, and throw expression into terms. In that way, and for that reason, singing brings us nearer to the most of our dumb friends, than articulation. Yet, if we constantly talk to animals as if they understood us, we shall find ourselves involuntarily modifying our sounds, and shall be surprised to find how much they evidently comprehend our meaning.

The relation which we assume to animals, modifies their articulation. It is not the parrot only that learns to talk from human intercourse, but all animals and birds as well. You have only to test this in your own yard, or on your own farm.

I have a horse that, when first purchased, knew human beings from some unpleasant side, and was accustomed to put on terrible pretences of devouring small boys. They had probably deserved to be eaten. She never vocalized, except a snort of anger, and declined any familiarity. She is now capable of expressing herself in a way that would satisfy Dean Swift. There is a consciousness in every motion that she is conversing with me; and she has quite a vocal range of affection and inquiry.

That this is capable of becoming a matter of heredity, you can determine in a short time. Try with a small herd of cows or a flock of sheep to breed for intelligence and expression, and you will find that your herd is even more capable of evolution and natural selection in the moral and emotional line, than in physical characteristics of a lower sort. I have had a breed of cows to the sixth generation. In only one or two crosses has this stock failed of peculiar mildness of disposition; in those exceptions the intelligence was more than ever marked, but with a tendency to trickery. But in all cases the evident desire to communicate and express emotions to human beings, has been predominant, and the ability has, I think, increased.

A Jersey cow very rarely lows, but has a range of soft kindly sounds for her human friends.

In all cases it is necessary to remember that animal language, begins on the level of emotional life; and in neither case rises often to the purely intellectual.

It is not impossible to meet men from some of the English counties, and the Irish, whose vocabulary does not exceed two or three hundred words. If we are to believe reports, Australians have fewer.

Articulation is a purely intellectual affair to cover ideas, unless used in the mimicry of parrots. The animal life does not require it. It requires only ejaculation and projected sounds, modified by passion or emotion.

Love, hate, anger and desire, are as plainly told in bird language, as in the machinery of human words. Indeed, the language of animals tells all these things more emphatically and gracefully. Words express love, only when they borrow the modulations of the dove.—*St. Louis Globe-Democrat.*

